# Statistics of Amputations in the New York Hospital, from January 1st, 1839, to January 1st, 1848

**Published:** 1849-01

**Authors:** Henry W. Buel

**Affiliations:** Resident Surgeon


### ARTICLE IV.

*Statistics of Amputations in the New York Hospital, from January 1st*, 1839, to *January 1st*, 1848. By Henry W. Buel, M. D., Resident Surgeon.

[The following summary is made up from tabular statements by the author, and gives the general results, which are highly interesting.]

It will be seen that the whole number of amputations here presented amounts to ninety-one; of which, twenty-six were fatal; making the mortality 28·57 per cent.

Of amputations at the hip-joint there was one, and that fatal.

Of amputations of the thigh, the whole number was thirty-four, of which ten were fatal; making the mortality 26·47 per cent.

At the knee-joint there was one amputation, and that fatal.

Of amputations of the leg, the whole number was twenty-four, of which seven were fatal; making the mortality 29·16 per cent.

Of amputations at the shoulder-joint, the whole number was nine; of which four were fatal; making the mortality 44·44 per cent.

Of amputations of the arm, the whole number was eleven, of which none were fatal.

Of amputations of the forearm, the whole number was thirteen; of which three were fatal; making the mortality 23·07 per cent.

So that we have sixty amputations of the lower extremity; of which nineteen were fatal; making the mortality 31 66 per cent.

While of thirty-three amputations of the upper extremity, seven were fatal; making the mortality 21·21 per cent.

With regard to the methods employed in these various operations, the reports of the cases do not, in every instance, specify the method; but in forty-nine cases (the whole number in which it is specified), twenty-four were performed by double-flap operation, and twenty-five by circular operation. Of the first number, four were fatal; making the mortality 17·66 per cent. Of the last, three; making the mortality 12 per cent.

Of the double-flap operations, there were fifteen of the thigh, of which two were fatal; three of the leg, of which one was fatal; four of the arm, and one of the forearm, of which none were fatal.

Of the twenty-five circular operations, six were of the thigh, of which none were fatal; fifteen were of the leg, of which three were fatal; three were of the arm, and one of the forearm, of which none were fatal.

Secondary hemorrhage occurred in only one of these forty nine cases, and in that, it occurred twice.

There were, in the whole number, two other cases, making, in all, three cases of secondary hemorrhage. The greater mortality of the flap operation may, perhaps, be attributed to the greater proportion of thigh amputations performed in that manner.

As to the question, whether primary or secondary amputation is preferable, it is evident, that an equal number of similar cases should be selected from each class, in order to institute anything like a just comparison. It is customary at the New York Hospital, when amputation is demanded after severe injuries, to operate before the accession of inflammatory action. So that, strictly speaking, the occasions for secondary amputations will be comparatively rare.

Of the whole number of amputations, sixty-two were the result of injuries, and were fatal in nineteen cases; making the mortality 30·64 per cent.

Of these, thirty-six were primary amputations; of which there were of the hip-joint one, and that fatal; of the thigh, seven, of which four were fatal; of the leg, twelve, of which five were fatal; of the arm, seven, and of the forearm, five, of which none were fatal. Making the mortality 27·77 per cent.

The remaining twenty-six amputations may all be said to have resulted from injuries of a more or less severe character; but, as will be seen, they were performed at very different periods after the original injury.

Of this number, eleven were amputations of the thigh, of which three were fatal. At the knee-joint, one, which was fatal; of the leg, seven, of which one was fatal. At the shoulder-joint, five, of which three were fatal; of the arm, one, and of the forearm, one, of which neither was fatal; making the mortality 30·76 per cent.

The number of operations for various chronic affections was twenty-nine, of which six were fatal. Of the thigh there were eighteen, of which four were fatal. Of the leg, five, of which none were fatal; of the arm, three, of which none were fatal; of the forearm, four, of which two were fatal; making the mortality 20·67 per cent.

The ages of the patients operated upon were as follows:—

| | | | | | |
|---|---|---|---|---|---|
| Under | 10 years of age, | 4, | of whom | 1 | died. |
| Between | 10 and 20, | 11, | " | 1 | " |
| " | 20 and 30, | 32, | " | 12 | " |
| " | 30 and 40, | 21, | " | 5 | " |
| " | 40 and 50, | 11, | " | 4 | " |
| " | 50 and 60, | 9, | " | 2 | " |
| " | 60 and 70, | 2, | " | 0 | " |
| " | 70 and 80, | 1, | " | 1 | " |
| | | 91 | | 26 | |

The whole number of females was nine, of whom three died.

Of the minor operations upon the lower extremity, during the same period of time, the following is a statement. There have been two partial amputations of the foot, through the articulation of the tarsus, and two through the metatarsal bones. In one of the latter cases, amputation through the whole five metatarsal bones was performed. In another, it was done through the first metatarsal bone. Disarticulation of the great toe was performed in thirteen instances, and amputation through one of the phalanges in two instances; of which operations in the great toe, ten were for frost-bites, and the others for injuries of various kinds. Disarticulation of one of the lesser toes in six instances, and through the phalanges in four; of which seven were for frost-bites, and three for injuries of various kinds.

Upon the upper extremity, amputation at the first carpo-metacarpal articulation in two instances, and, in one instance, the second and third; in another, the third and fourth; in another, the third, fourth, and fifth; and in another, the fifth metacarpal bones were removed at this articulation. The thumb was amputated at the metacarpo-phalangeal articulation, in five instances; through the first phalanx in one, and at the phalangeal articulation in one instance. One patient had amputation performed through three of the metacarpal bones of the fingers; another, through two; and three others, each through one of those bones. Amputation of the finger was performed at the metacarpo-phalangeal articulation in twenty instances; through the first phalanx in one instance; at the first phalangeal articulation in five instances; through the second phalanx in one; at the second phalangeal articulation in five instances, and through the last phalanx in one instance. Seventeen of these operations were for gun-shot wounds. None of the minor operations, upon either extremity, proved fatal.

Such being the facts in regard to the mortality of these operations, a word concerning the causes which have produced it. Mr. Phillips attributes the increased mortality in the city of London to the greater nember of railroad accidents that have happened within a few years past, and the same cause has undoubtedly produced a similar effect here. It will be readily admitted that this class of accidents presents some of the most formidable, or even desperate cases in which amputation is ever demanded. In these cases the injury on account of which the operation is indicated, is most commonly attended with others of a serious character. Such patients, also, are not only very frequently without surgical attendance for many hours, and even for days, but are subject to the additional hazard of transportation from a very considerable distance. Under this head might also be included a similar, and not uncommon class of accidents occurring at the landings of ferries. Making a distinct class of these injuries, it will be found that thirteen of the capital amputations above mentioned, were the result of such casualties, and no less than six of them unsuccessful.

Another class of accidents, which presents equally desperate cases, is that of extensive burns. Every one is aware of the great mortality which attends these accidents, where a great extent of surface is involved. Six amputations were performed for this cause. Two of the patients were males, and four were females; of which number three females died. Here, then, are two classes of amputations, in each of which, the mortality is nearly fifty per cent.

Two patients died of phthisis, after amputation of the forearm had been performed, for scrofulous disease of long standing. Two patients died of tetanus, one upon the ninth, and the other upon the seventh day, after amputation at the shoulder joint.

Four amputations were performed for malignant disease; of this number, three were so far cured, as to leave the hospital with a fair chance of reprieve for some time longer. One case was fatal; the disease in that instance attacking the lungs after the patient had so far recovered from the amputation, as to be up, and about his ward. In the successful cases, the recovery seems to have been very rapid.

Fourteen amputations of the thigh were performed for disease of the knee-joint. Only two of these cases were fatal; the patients in both instances being black men. Including those just mentioned, four of the patients operated upon were blacks, and of these, three died; a fact which, so far as it goes, tends to corroborate the common impression that amputation, or any severe operation, is not as well borne by blacks, as by whites.

By far the most common cause of the minor operations upon the upper extremity, was gunshot wounds, and upon the lower extremity, exposure to cold.

On the whole, then, though the mortality after capital operations of this class appears at first sight very great, it is impossible to avoid the conclusion that this result occurs rather *in spite*, than *on account*, of the operation itself. To adopt a contrary opinion, would be as rational as to condemn any agent in the materia medica proper, on account of the mortality which might happen, at any period after its administration.

The results obtained above, are similar in character to those presented by Drs. Norris and Hayward, in their respective papers upon this subject. In some particulars, they are less favorable than theirs, in others more so; accurate estimates, however, can only be formed from a large number of cases.

It would have been interesting, if those gentlemen had made separate classes of the amputations at the different joints, so as to show their comparative danger.

The very great mortality attending this class of operations in the Parisian hospitals, renders such information particularly desirable. Dr. Norris, however, mentions several amputations "at the joints," of which four were cured, and three died. He has given a record of eighty amputations performed upon seventy-nine patients during ten years; while Dr. Hayward has reported seventy amputations performed upon sixty-seven patients during about eighteen years. So that we have an aggregate of two hundred and forty-one operations performed on two hundred and thirty-seven patients in civil hospitals in the United States, and under circumstances as nearly similar as we could expect to obtain. A brief summary of these data with regard to two or three of the more important points, is herewith presented:

| | Philadelphia. | Boston. | N. York. | Total. |
|---|---|---|---|---|
| Whole number | 79 | 67 | 91 | 237 |
| Cured | 57 | 52 | 65 | 174 |
| Mortality per cent. | 27·84 | 22·38 | 28·57 | 26·58 |
| Upper extremity | 32 | 10 | 33 | 75 |
| Cured | 27 | 9 | 26 | 62 |
| Mortality | 15·62 | 10 | 21·21 | 12·73 |
| Lower extremity | 47 | 57 | 60 | 164 |
| Cured | 31 | 43 | 41 | 115 |
| Mortality | 34·04 | 24·55 | 31·66 | 29·87 |
| For chronic disease | 25 | 45 | 29 | 99 |
| Cured | 21 | 39 | 23 | 83 |
| Mortality | 16 | 13·33 | 20·68 | 16·16 |
| For injuries | 54 | 22 | 62 | 138 |
| Cured | 37 | 12 | 43 | 92 |
| Mortality | 31·48 | 45·45 | 30·64 | 33·33 |

In the foregoing table, it will be seen that the numbers given, are those of the patients operated upon. The comparison might be extended to other points, but it is unnecessary to do so at this time.

The results here shown are not discreditable to American surgery, as compared with European. Mr. Phillips gives the following, as a statement of the cases collected by him:

| | | | | |
|---|---|---|---|---|
| In France | 203 cases | 47 deaths, or | 23·10 per cent. |
| In Germany | 109 " | 26 " | 23·85 " |
| In America | 95 " | 24 " | 25·26 " |
| In Great Britain | 233 " | 54 " | 22·66 " |

These statistics were obtained from a great variety of sources, by Mr. Phillips. He does not particularize the sources of his information with regard to America. Those in Great

Britain were "drawn from a large number of hospitals, and from the *private practice of hospital surgeons*, selections being made, so as to render the result shown, a fair representation of what actually occurs."

Looking at single places, we find that,

For London, the cases amount to 107, and the deaths to 28, or 26·16 per cent.

For Paris, according to M. Malgaigne, the cases amount to 560, the deaths to 299, or 53·39 per cent.

For three of our principal cities as given above, the cases amount to 237, the deaths to 53, or 26·58 per cent.—*Am. Jour.*

